# Acknowledgment to the Reviewers of *Gels* in 2022

**DOI:** 10.3390/gels9020079

**Published:** 2023-01-18

**Authors:** Gels Editorial Office

**Affiliations:** MDPI AG, St. Alban-Anlage 66, 4052 Basel, Switzerland

High-quality academic publishing is built on rigorous peer review. *Gels* was able to uphold its high standards for published papers due to the outstanding efforts of our reviewers. Thanks to the efforts of our reviewers in 2022, the median time to first decision was 12 days and the median time to publication was 31 days. Regardless of whether the articles they examined were ultimately published, the editors would like to express their appreciation and thank the following reviewers for the time and dedication that they have shown *Gels*:
Abang Zaidel, Dayang NorulfairuzLi, YueshengAbbasi Moud, ArefLiao, JinfengAbd El-Fattah, AhmedLigorio, CosimoAbd Mutalib, Noor AziraLim, Eun-KyungAbdallah, MarwaLim, VuanghaoAbdellatif, Mohamed MehawedLin, Chun-RongAbd-elsalam, Wessam H.Lin, Hung-YinAbdi, GholamrezaLin, ShiruAbdolahinia, Elaheh DalirLin, WeifengAbdul Rahman, Ahmad TaufekLin, Zuan-TaoAbdullah, C. K.Lindvold, LarsAbdullah, Mohd Mustafa Al BakriLing, IreneAbdulridha, Wasna’a M.Linklater, DenverAbdurrahman, MuslimLinul, EmanoilAboud, Heba M.Lisignoli, GinaAboudzadeh, M. AliLiu, JieAbudula, TuerdimaimaitiLiu, QiangAbu-Reziq, RaedLiu, TiancaiAcar, NurayLiu, Xian HuAcik, GökhanLiu, XiuyingAcimovic, MilicaLiu, YangzhenAcuña, Sergio M.Liu, Ying DanAguirre-Álvarez, GabrielLiu, YonglanAhmad, Mohammad Norazmi BinLiu, ZhangAhmad, WaqasLochab, BimleshAhmadian, ElhamLomova, MariaAhmed, AbdelaalLopes, João HenriqueAhmed, Mohammed MuqtaderLópez Franco, YolandaAhmed, SahnawazLopez-Maldonado, Eduardo A.Ahmedova, AnifeLou, JunzheAhn, Chi BumLouis, FionaAjayaghosh, AyappanpillaiLozano, DemetrioAkazawa, HiroyukiLozano-Torres, BeatrizAkhtar, Md JawaidLozinsky, VladimirAkilbekova, DanaLozynskyi, VasylAkimasa, SenoLuckanagul, Jittima AmieAl-Anssari, SarmadLufrano, FrancescoAlavi, MehranLuhar, SalmabanuAlbukhaty, SalimLuna, Juliana MouraAlcudia, AnaLuo, YongxiangAlencar, Éverton N.Ma, YutianAlexandrino Júnior, FranciscoMädler, LutzAlexopoulos, Alexandros H.Magkoev, Tamerlan T.Ali, FayazMahato, NeelimaAli, GhulamMahendradatta, MetaAli, HaiderMahon, Clare S.Ali, Jagar A.Maiti, BinoyAli, NoorshidaMaji, SamarendraAli, SalmanMakarov, Vladimir IgorevichAlias, EkramMaleki-Ghaleh, HosseinAliberti, AnnaMalekmohammadi, SamiraAlijagić, AndiMalik, SudipAlipour, AbdolmajidMalkin, AlexanderAlmásy, LászlóMallamace, FrancescoAlmoshari, YosifMallandrich, MireiaAl-Musawi, SharafaldinMalvandi, Amir MohammadAlmutairi, Fahad M.Malviya, NovinaAl-Odayni, Abdel-BasitManikkath, JyothsnaAlonso-Moreno, CarlosManjunatha, Jamballi G.Al-Sabagh, Ahmed M.Manzar, Mohammad SaoodAlshammari, Abdullah S.Manzoor, Muhammad FaisalAltunbek, MineMaras, PiotrAlves, PatríciaMarcuello, CarlosAmara, AmroMargeta, KarmenAmico, GiandomenicoMarien, YoshiAmin, AmrMarino, MicheleAminian, AliMarques, IsabelAmir, ZulhelmiMarrale, MaurizioAmirthalingam, SivashanmugamMarszalek, Jolanta ElzbietaAmiza, Mat AminMartín-Alfonso, Jose EnriqueAmmirati, MarioMartinelli, ChiaraAmombo Noa, Francoise M.Martinho, Fernando C. G.Amran, MugahedMasruchin, NanangAnastasescu, CrinaMatczuk, MagdalenaAndrade, Diogo E. V.Mattea, FacundoAndrés, AnaMaykel, González-TorresAndronoiu, DoinaMazumder, NirmalAngelov, BorislavMazyed, EmanAnsari, Mohammad JavedMba Blázquez, MiriamAntić-Stanković, JelenaMd Isa, Illyas BinAntosik, AdrianMedarevic, DjordjeAntunes, Joana C.Mehandzhiyski, AleksandarApostolova, Margarita D.Mehrali, MehdiAqad, EmadMehta, Naresh KumarArce, Florencio Jr.Mei, XuanArellano-Garcia, Maria EvaristaMellati, AmirArenillas, AnaMelnikova, NinaArif, MuhammadMemon, AsadullahAriga, KatsuhikoMenichetti, LucaArinstein, ArkadiiMésini, Philippe J.Arkas, MichaelMiao, XiaoqingArlyapov, Vyacheslav A.Micutz, MarinArora, DeepikaMielańczyk, AnnaArtusio, FioraMilakin, Konstantin A.Asa’ad, FarahMilcovich, GesmiAsasutjarit, RathaponMilojević-Rakić, MajaAsefnejad, AzadehMiotke-Wasilczyk, MartaAshames, AkramMirza, AamirAshfaq, MohammadMirza, SabiruddinAshish, Deepankar KumarMishra, KuldeepAssadi, AymenMitoma, YoshiharuAsyraf, Muhammad Rizal MuhammadMo, GaomingAvetissov, IgorMocioiu, Oana CătălinaAwad, SameerModibane, Kwena DesmondAwual, Md. RabiulMofidfar, MohammadAydemir, LeventMohamadian, NimaAydin Kose, FadimeMohamed, M. SheikhAziz, Ikmal HakemMohamed, Mohamed GamalBabaei-Ghazvini, AminMohammadi Samani, SoleimanBadawi, Ahmed K.Mohammadi, ShookaBadilli, UlyaMohan Ganesh, G.Badr, AhmedMohidem, Nur AtikahBadraoui, RiadhMohiuddin, OmairBadshah, Syed LalMohyeldin, Mohamed SamirBahramian, Ahmad RezaMollica, FrancescoBahsis, LahoucineMomekova, Denitsa B.Bai, BaojunMonique Tohoué, TognonviBai, ChengyingMonjo, MartaBai, YingruiMonroy-Guzman, FabiolaBaidya, AvijitMontassar Aidi, SharifBains, AartiMoradi, SaraBakhsheshi-Rad, Hamid RezaMorazzoni, FrancaBalamurugan, RathinamMorgado, JorgeBaldino, LuciaMorgat, ClémentBall, VincentMorimoto, NobuyukiBallabh, AmarMorocho-Jácome, Ana LucíaBandarenka, Hanna V.Moscone, DanilaBanerjee, BubunMoses, JeyanBar, JuliaMosivand, SabaBardakci, FevziMostovaya, Olga A.Bari, EliaMounir, HassaniBarišić, VeronikaMousa, Hamouda M.Barros, JoanaMousavi Nejad, ZohreBartzas, GeorgiosMousavi, Seyyed MojtabaBashir Yahya, EsamMoutsatsos, ArgyrisBastiat, GuillaumeMu, QifengBayan, MohammadMudgel, PritiBecerra-Bayona, SilviaMuhammad, AkhlaqBedilo, AlexanderMuhammad, Ida IdayuBehravan, AmirMukai, MasaruBelchior, AnaMulla, Sikandar I.Benetatos, PanayotisMunoz-Pinto, DanyBengoechea, CarlosMuranov, KonstantinBenzarti, ZohraMurshid, NimerBertolo, Mirella Romanelli VicenteMurto, PetriBeskopylny, AlexeyMusa, ArafaBezhin, NikolayMusić, SvetozarBhalani, DixitMussel, MatanBhatia, DhirajMustapa, Ana N.Bhatt, AnugyaMuthupandian, SaravananBhattacharjee, SouravNaccache, Mônica FeijóBhattacharya, SankhaNadeem, MuhammadBhingaradiya, NutanNadhman, AkhtarBhupathyraaj, MullaicharamNagai, DaisukeBhuvaneswari, VenkateswaranNagaraj, RamakrishnanBidarra, Sílvia J.Nagarajaganesh, B.Bielan, ZuzannaNagarajan, SubbiahBikiaris, DimitriosNagdalian, AndreyBinti Mohd Nadzir, MasrinaNakahata, MasakiBintinger, JohannesNarayanan, AmalBîru, Elena IulianaNathanael, Arputharaj JosephBispo-Jr, Airton GermanoNawab, YasirBiswas, SantidanNawaz, AsifBoddu, Sai H. S.Nayak, RatiBoddupalli, AnuraagNazir, AkmalBogdan, NeamtuNazir, ImranBohdal, LukaszNechifor, GheorgheBojin, Florina M.Neela, SatheeshBokias, GeorgiosNeffati, RiadhBolla, GeethaNelson, Arif Z.Bonardd, SebastianNemes, DánielBonetti, LorenzoNenadovic, SnezanaBoni, FernandaNeto, MariaBoothby, JenniferNežić, LanaBorcan, FlorinNg, Eng-PohBorges, João PauloNg, Wei LongBormashenko, EdwardNguyen, SonBradbury, PetaNicoletta, Fiore PasqualeBrambilla, ElisaNicolucci, PatríciaBratskaya, SvetlanaNicu, RalucaBrnčić, Suzana RimacNie, YiBrocca, StefaniaNiknezhad, Seyyed VahidBrushi, Marcos LucianoNikolaychuk, Pavel AnatolyevichBryja, LeszekNingrum, AndriatiBrzyski, PrzemysławNingrum, Eva OktaviaBucatariu, Sanda-MariaNistor, AlexandraBucciarelli, AlessioNitti, PaolaBuchaim, Rogério LeoneNiu, WantingBuj-Corral, IreneNjimou, Jacques RomainBullón, JohnnyNoreen, SobiaButruk-Raszeja, BeataNowaczyk, JacekButu, MarianNowoświat, ArturBuyong, Muhamad RamdzanÓ Conghaile, PeterCaddeo, FrancescoO’Connor, Naphtali A.Caicedo, CarolinaObolewski, KrystianCalzada, FernandoOchi, RikaCametti, CesareOgórek, RafałČanadanović Brunet, JasnaOhsedo, YutakaCanciani, ElenaOkesola, BabatundeCandiota, AnaOkonogi, SiripornCanevali, CarmenOlaru, DumitruCao, YifengOlaru, Octavian TudorelCaprarescu, SimonaOlojede, Ayoyinka O.Cârâc, GetaOmachi, HarukaCarlos Salay, LuizOnani, Martin O.Carmona, NoemiOnu, Chijioke ElijahCarmona-Ribeiro, Ana M.Orakdogen, NerminCarroccio, Sabrina CarolaOrion, ItzhakCarvalho, SilviaOrnelas, CatiaCasarejos, EnriqueOrsini, GiovannaCastaño, JohannaOrtega, Miguel A.Catucci, LuciaOrtega-Lara, WendyÇelebi-Saltik, BetülOssowicz, PaulaCelesti, ConsueloOsterne, ViníciusCerbone, LuigiOstos, Francisco JoséCerqueira, Miguel ÂngeloOtsuka, HidenoriCervellati, FrancoOu, ZihaoChaijan, ManatOueslati, WalidChakraborty, PriyadarshiOvais, MuhammadChalkias, Dimitris A.Owais, MohammadChan, Chin HanOzaltin, KadirChan, Ming HsienÖzdemir, Zeynep GüvenChandel, Arvind SinghÖzgür, AykutChang, Ying PingOzon, Emma AdrianaChantarak, SirinyaÖztürk, MuhittinChaosap, ChanpornPacheco, NeithChauhan, Ghanshyam SinghPaladini, GiuseppeChauhan, Narendra Pal SinghPalaniappan, ArunkumarChavali, MurthyPalma, Paulo J.Chen, DongPaltanea, VeronicaChen, GuiliangPan, HouwenChen, JingsiPanda, DibyaChen, MingfuPanda, Pradeep KumarChen, QiangPandele, MadalinaChen, QuanPandurangan, PrakashChen, ShikunPang, Yean LingChen, WeiPanomsuwan, GasiditChen, Wen-ChengPanthi, KrishnaChen, XiangzhongPantić, MilicaChen, YiPantuso, ElviraCheng, Chi-AnPanzarasa, GuidoCheng, Chih-yuPapadakis, RaffaelloCheng, HanlinParaskevopoulou, PatrinaChew, Khian-HooiParayangattil Jyothibasu, JincyChiang, Chang-YuePardeshi, SagarChiang, Tzu HsuanPardo, AlbertoChircov, CristinaParekh, DishitChiriac, AuricaPark, Hyung-HoChirinos, RosanaPark, InmyoungChittasupho, ChudaPark, Jung-hyunChiulan, IoanaParlatescu, IoaninaChoi, SejinParmaksiz, MahmutChou, Shih-FengParvinzadeh Gashti, MazeyarChoudhary, NishaPashuck, E. ThomasChow, Tsz HimPasinszki, TiboChuan, Lee TePatel, GayatriCiolacu, DianaPatra, BishnubrataCiolacu, FlorinPattanayek, Sudip K.Civera, Maria ConcepciónPaucean, AdrianaČolović, BožanaPauer, WernerConti, AllegraPaxton, Jennifer Z.Contini, ClaudiaPedzich, ZbigniewCórdoba-Diaz, ManuelPelegrino, Milena TrevisanCorrias, AnnaPeng, QiongyaoCoseri, SergiuPeptu, CristianCosta, Mirian Cristina GomesPereira, Patrícia RibeiroCrosby, CodyPereira, TatianaCruz, Juan C.Perez, SergeCuevas Bernardino, Juan CarlosPérez-Larios, AlejandroCunha, Eunice P. F.Permana, Andi DianCurreli, NicolaPervaiz, MuhammadDa’na, EnshirahPessoa, Rodrigo SávioDaege, Haile FentahunPhaechamud, ThawatchaiDagdag, OmarPham, Tien DucDai, TaotaoPhan, Giang V. H.Damrongsakkul, SiripornPhungamngoen, ChanthimaDan, DobrotaPiattelli, AdrianoDanar, PraseptianggaPicciani, Paulo H. S.Daniel, LikiusPiluso, SusannaDaniel, SobhiPino-Ramos, Victor H.Daniele, MoroPippa, NatassaDapcevic, TamaraPisano, RobertoDargaville, Bronwin L.Pisarski, WojciechDas Chagas, RafaelPishavar, ElhamDas, ApurbaPlatta, HaraldDas, ProlayPławińska-Czarnak, JoannaDas, Raunak KumarPoater, AlbertDastgeer, GhulamPongjanyakul, ThanedDatt, GopalPopa, CatalinDatta, DebikaPopa, LacramioaraDávila-Pulido, Gloria I.Popa, Vlad T.Davoodi, ShadfarPopescu, IrinaDe Araújo, Daniele RibeiroPopović-Bijelić, Ana D.De Giglio, ElviraPotapov, Vadim V.De Oliveira Filho, Josemar GonçalvesPothan, LalyDe Oliveira, Ariel ColaçoPourmohammadi, KianaDe Paula Santos, Luis FelipePourshahrestani, SaraDe Souza Loureiro, ElisângelaPrakash, M. DurgaDe Souza, Aline GomesPramanik, BapanDe Souza, Paulo RicardoPrapainainar, PaweenaDe Zorzi, RitaPrasad Tiwari, ArjunDel Prado Audelo, María LuisaPrasad, AlishaDelgado-Charro, M. BegoñaPraveen, VakayilDemirtaş, Tuğrul TolgaPrieto, GerardoDeng, Ming JayPrikhozhdenko, EkaterinaDeon, MoniquePrimi, RiccardoDetsch, RainerProkopijević, MilošDev, AbhimanyuPucek, AgataDeveci, HuseyinPuia, CosminDhaliwal, Gurjot S.Puiggalí, JordiDi Chenna, PabloPuišo, JuditaDi Pierro, ProsperoPuri, VinamDi Stefano, BarbaraPurohit, Shiv DuttDiaferia, CarloPutro, Jindrayani NyooDíaz-Montes, ElsaPutz, Ana MariaDiego, Juan García-BernaltQiang, LiDinca, ValentinaQu, MingDinescu, SorinaQu, XiaozhongDing, ShujiangQushawy, Mona K. E.Dini, GhasemRabaeh, KhalidDinis, Maria Alzira PimentaRacoviceanu, RoxanaDinita, AlinRadhouani, HajerDirè, SandraRadu, Ionut-CristianDojcilovic, RadovanRadu, Nicoleta NicoleDolak, İbrahiimRadwan, Ahmed E.Domalik-Pyzik, PatrycjaRafael Jesus Gonçalves, RubiraDomenici, FabioRafiq, SerwanDong, RuihuaRaghavendra, NaveenDorcioman, GabrielaRaghu, Anjanapura V.Dos Santos, Elisabete PereiraRahdar, AbbasDrabczyk, AnnaRahman, Hafiz Muhammad Abd UrDu, PengRahman, Mohammed M.Duc, CarolineRai, AmitDudás, ZoltánRailean-Plugaru, VioricaDurairaj, KaliannanRaja, Iruthayapandi SelestinDurán-Álvarez, Juan CarlosRajakumar, GovindasamyDurgun, Meltem EzgiRamachandran, Sinija VadakkeppulparaDušková-Smrčková, MiroslavaRamalho, Maria JoãoDuteanu, NarcisRamanauskienė, KristinaDyl, TomaszRamedani, ArashDymond, MarcusRamesh, KalyanDyszkiewicz-Konwińska, MartaRameshbabu, Arun PrabhuEkielski, AdamRamirez Jimenez, AlejandroEl Rasssy, HoussamRana, DipakElena, HusanuRana, Jahanban-EsfahlanEligiusz, PostekRangelov, Stanislav M.Elkhenany, HodaRanjan Rauta, PradiptaEllafi, AbdulazizRao, K. S. V. KrishnaElma, MuthiaRao, Kummara MadhusudanaElmanovich, IgorRao, KummariElnahas, Hanan M.Raschip, Irina ElenaElouarzaki, KamalRasheed, MohammedElsebaie, Essam MohamedRashid, ZerminaElsewedy, HebaRaspanti, MarioEmran, MohammedRasul, AkhtarEscobar-Barrios, Vladimir AlonsoRatke, LorenzEsfe, MohammadRau, IleanaEsposito, TizianaRaut, AshwinEstevez, Juan C.Raza, FaisalEthica, Stalis NormaRazavi, RaziehExner, Ginka K.Reczek, LidiaEyadeh, MolhamReczyńska, KatarzynaEzgi, PulatsuReece, Lisa M.Fábián, IstvánRehan, Zulfiqar AhmadFaggian, AlessiaRehman, WajidFahim, Irene SamyRen, RongguoFaivre, AnneliseRéthoré, GildasFang, JiyuRevin, Viktor V.Farag, Mayada RagabRezvani, Ali RezaFarrukh, AleezaRhazi, MohammedFatemi, MobeenRiess, GisbertFatimi, AhmedRinoldi, ChiaraFatoni, AminRistic, IvanFediuk, RomanRiva, LauraFernández-Quiroz, DanielRizvi, Syed A. A.Ferreira, FernandoRizwan, MuhammadFerreira, Paula M.Rodrigues, Márcia T.Ferreira-Neto, Elias PaivaRodríguez, Rusbel ConeoFerretti, ElenaRodríguez, Sergio A.Fidan, IsmailRosseto, HélenFigueroa López, Kelly JohanaRossi, FilippoFilarowski, AleksanderRossi-Marquez, GiovannaFilip, PetrRoudaut, GaelleFilippini, FrancescoRoy, SwarupFiorica, CalogeroRozas, RobertoFlores-Colen, InesRubio, JuanFlorowska, AnnaRusen, EdinaFoglia, FabriziaRusso, FrancescaFonseca-Garcia, AbrilRusso, TeresaFouad, ShahinazeRusu, Alina GabrielaFox, NiamhRydzak, LeszekFrancesco, DecataldoRydzek, GaulthierFranco, Camilo AndresRyplida, BennyFranco, InmaculadaSachdev, AbhayFreitas, Ana IsabelSaeed, ShaukatFrielinghaus, HenrichSafdarian, MehdiFröhlich, EleonoreSaffarionpour, ShimaFu, Lian-HuaSagiri, SaiFu, ShaozhiSaha, RahulFu, WenxinSahiner, MehtapFu, YongzhuSahoo, PathikGabriela, IsopencuSakai, TakamasaGac, Jakub M.Sakowicz- Burkiewicz, MonikaGad, ShadeedSakthivel, KogularasuGadomska-Gajadhur, AgnieszkaSalahuddin, Bidita BinteGaeta, MassimilianoSaldias, CesarGafurov, MaratSaleem, MuhammadGahruie, Hadi HashemiSaleh, BahramGajendiran, ManiSaleh, Mohamed A.Gajtko, OlgaSaleh, TawfikGaladari, HassanSaliev, TimurGalangau, OlivierSalimi-Kenari, HamedGallardo, AlbertoSalomatina, Oksana V.Gallegos, AlbertoSalvia, MichelleGalli, CarloSamal, SangramGallo, GiovanniSambataro, SergioGallo, NunziaSamhan-Arias, AlejandroGallo, SalvatoreSan Andrés, María PazGambelli, AlbertoSánchez Aldana, DanielaGan, ZongsongSantacruz Ortega, Hisila Del CarmenGangacharyulu, DasarojuSantos, Francisco Eroni Paz DosGangrade, AnkitSantos, João H. P. M.Gao, JieSaperas, NuriaGao, MengSaraydın, DursunGaponenko, Nikolai V.Sarbu, AndreiGaravand, FarhadSargazi, GhasemGarcía Ramírez, Mario AlbertoSari Yilmaz, MügeGarcia-Gonzales, CarlosSarıcaoğlu, Furkan TürkerGarcía-Muñoz Rodrigo, FerminSarosi, CodrutaGarcía-Sifuentes, Celia OliviaSata, NorikoGarg, AnujSatapathy, SitakantaGarg, RajniSathirakul, KorbthamGarncarek, ZbigniewSato, TakeshiGbadamosi, AfeezSavaşkan Yilmaz, SevilGe, GangSavoji, HoumanGentili, DenisSaw, Phei ErGeorge, DhanyaSawai, JunGeorgiyants, Victoriya A.Saxena, Kuldeep K.Getachew, GirumScandurra, AntoninoGhazwani, MohammedSchoenhagen, PaulGhiasi, FatemehSchweitzer-Stenner, ReinhardGhiass, Mohammad AdelScialla, StefaniaGhosh, KumareshSeabra, CatarinaGhosh, SantanuSecuianu, CatincaGhosh, SougataSefat, FarshidGiannakas, ArisSeifzadeh, DavodGieroba, BarbaraSelvam, KandasamyGierszewska, MagdalenaSengupta, PoulomiGiram, PrabhanjanSenses, ErkanGiuri, DemetraSenthil, RethinamGlukhova, Olga E.Serikov, ArkadyGniewosz, MałgorzataSerrano-Aroca, ÁngelGok, OzgulSethi, GautamGołaszewski, JacekSgreccia, EmanuelaGolubeva, ElenaShafiq, IqrashGomez, JovannyShafiq, ZahidGomez-Pinedo, UlisesShafqat, Syed SalmanGonçalves, Elsa MargaridaShah, TejalGong, PeiweiShahabuddin, SyedGonzález-Reza, RicardoShahid, M.Goonoo, NowsheenShahidi, Seyed-AhmadGorshkova, NataliaShaik, Mohammed RafiGotoh, HiroakiShakeel, FaiyazGovindan, RajiShalygin, AntonGrabska-Zielińska, SylwiaShamsabadi, Elyas AsadiGreco, AntonioShankar, SarojiniGrigoraş, Cristina GabrielaShariati, MahdiGrinberg, Valerij YaSharifi, EsmaeelGrumezescu, Alexandru MihaiSharker, Shazid Md.Grza̧dka, ElzbietaSharma, Amit KumarGuenet, Jean-MichelSharma, AnkurGuerra-Sanchez, GuadalupeSharma, KashmaGui, YingangSharma, NitinGuillen-Grima, FranciscoSharma, ShubhamGulbake, ArvindSharonova, Olga M.Gulicovski, JelenaShenderovich, Ilya G.Gull, NafisaSheng, LiyuanGultekinoglu, MerveSheydaei, MiladGunji, TakahiroShi, WenGuo, DandanShi, YanshuGuo, HuShiekh, KhursheedGuo, SijinShimanouchi, ToshinoriGuo, WenwenShimoga, GaneshGuo, YanchuanShokry, HassanGuo, YouhongShrivastava, SajalGupta, CharuSievänen, ElinaGupta, PawanSikandar, Muhammad AliGupta, RajeevSilva, Cecy MartinsGupta, Ravi KantSilvestre, WendelGupta, SumeetSing, Swee LeongGurgen, SelimSingh, InderbirGus’kov, VladimirSingh, KamalendraGuzel Kaya, GulcihanSingh, Rajkumar SunilGyarmati, BenjáminSingh, SumanHabib, AhsanSingh, Yogendra PratapHabuda-Stanic, MirnaSingha, Nayan RanjanHadjighassem, MahmoudrezaSiregar, Januar ParlaunganHadjikakou, SotirisSivasamy, RameshHagio, TakeshiSivokhin, AlexeyHaider, Adawiya J.Skopinska-Wisniewska, JoannaHaimhoffer, ÁdámSkripkin, Mikhail YurievichHamadi, Naoufel BenSlovak, VaclavHamza, Mohammed F.Smith, Paul F.Han, HanSmith, Vincent J.Han, WenboSmułek, WojciechHanawa, TakehisaSnežana, PapovićHanks, TimothySöderberg, Daniel L.Hao, ChenSoliman, YasserHao, JinsongSoltani, MostafaHaponiuk, JózefSong, YounghanHaq, Muhammad AbdulSong, YuanhuiHarrington, DanielSong, YujunHasan, MurtazaSonker, Rakesh KumarHasan, NurhasniSopyan, IyanHashidzume, AkihitoSoromenho, Mário R. C.Hashimoto, MasanoriSosa, Filipe Hobi BordónHashimoto, YoshihideSoto Simental, SergioHassan, Adeel A.Spasova, MariyaHassan, AmjedSpoială, AngelaHassan, Mohamed A.Sreekanth Reddy, ObireddyHasturk, OnurSridhar, T. M.Hathout, RaniaSrikulkit, KaweeHe, Wan-LiSriram, BalasubramanianHe, Wei-DongSrivastava, AnirudhHedayati, SaraSrivastava, SamanvayaHedeșiu, MihaelaStanciu, Magdalena CristinaHeller, JanStankovic, BranislavHelsch, GundulaStanley, JohnHendrik, BargelStaś, MonikaHenrich, DirkStastny, PremyslHernández-Estrada, Zorba JosuéStella, GiuseppeHo, JamesSu, BenlongHong, Min-HoSukul, Pradip Kr.Hornok, ViktóriaSulimova, Maria A.Hosny, RashaSumarmono, JuniHosseinkhani, HosseinSumer, TayserHoward, MichaelSun, BoHrnjak-Murgić, ZlataSun, HeHsu, Che-JungSun, HuiHu, ChengSun, ShaolongHu, FangSun, Tao LinHu, JunhuaSunarwidhi, AnggitHu, YangSuner, Selin SagbasHua, MutianSuntornnond, RatimaHuan, SiqiSurayot, UtoompornHuanbutta, KampanartSurmenev, RomanHuang, Chun-YingSuta, Lenuta-MariaHuang, JinSutar, PapriHuang, ZehuanSzekely, GyorgyHuang, ZhengweiSzeleszczuk, ŁukaszHuda, NurulSzultka-Młyńska, MałgorzataHussain, SameerSzustakiewicz, KonradHussein, IbnelwaleedSzwast, MaciejIacob, AndreeaTadesse, F. TeferraIatridi, ZacharoulaTagami, TatsuakiIatrou, HermisTaghikhani, VahidIbrahim, NaimahTaher Yousefi, AmiriIglesias, EmiliaTajbaksh, MahmoodIglesias-Martínez, EmiliaTalebi, ZahraIgnjatovic, NenadTanaka, TomonariIhsanullah, IhsanullahTang, FushanIjima, HiroyukiTanhaei, BaharehIkada, YoshitoTarawneh, Ola A.Ikram, RabiaTarhan, OzgurIlg, PatrickTaya, Sofyan A.Ilic, NebojsaTenório-Neto, Ernandes TaveiraIlic-Stojanovic, SnezanaTeow, Sin-YeangIlyin, SergeyTeramoto, NaozumiImran, Abu BinTerraza, Claudio A.Inbaraj, Baskaran StephenThach, Ut DongInbraj, StephenThakur, SachinIngavle, GaneshTheivasanthi, ThirugnanasambandanIngole, PravinThiemann, ThiesIñiguez-Moreno, MaricarmenTian, WeiguoIonita, GabrielaTiggelaar, Roald M.Iqhrammullah, MuhammadTimmins, PeterIsaad, JalalToktarbay, ZhexenbekIshwarya, S. PadmaTomić, JelenaIslam, Md RashadulTopcu, Aykut ArifIsmadji, SuryadiTorres Pabón, Norma SofíaIsmail, AznanTounsi, AbdelouahedIspas-Szabo, PompiliaTouraud, DidierIstrate, BogdanTovar Rodríguez, Clara AsunciónIto, AkitakaTrajkovska Petkoska, AnkaIuliano, MauroTran, Ngoc QuyenJabbar, MohannadTreviño, EstherJaidev Chakka, Leela RaghavaTroncoso, ElizabethJain, Gaurav KumarTrotta, FrancescoJaipakdee, NapaphakTrubiani, OrianaJakubczyk, EwaTrucillo, PaoloJakubowski, WitoldTruong, Minh-DungJamil, Noorina HidayuTsou, Yung-HaoJammalamadaka, UdayTuǧcu-Demiröz, FatmanurJamnongkan, TongsaiTulay, OzcanJamshidi, ParastooTuleu, CatherineJan, Jeng-ShiungTurek, ArturJanarthanan, GopinathanTverdokhlebov, SergeiJantrawut, PensakTvrdy, KevinJardiel, TeresaTyliszczak, BożenaJaved, HayateUddin, FakharJawad, Ali H.Uhrynowski, WitoldJayash, Soher NagiUl Haq, EshanJedlovszky-Hajdu, AngelaUllah, AmanJerković, IgorUllah, MuhammadJerzykiewicz, LucjanUllah, RafiJeyalakshmi, RamaswamyUsai, PaoloJha, HaritUthaman, AryaJi, XiaofanUthappa, Uluvangada ThammaiahJia, ManpingValado, AnaJiang, ChengminValdez, BenjamínJiang, ShaohuaValente, JoanaJiang, ZhuoranVankayala, RavirajJilani, AsimVáradi, JuditJin, ZhengyuVasconcelos, Niedja FittipaldiJoão, SilvaVasilevskaya, ValentinaJohn Jervis, PeterVasquez, Magdaleno Jr.Johnson, RenjithVedhanarayanan, BalaramanJooybar, ElahehVeerasubramanian, Praveen KrishnaJoshi, Mahesh KumarVega, AlejandroJoshi, Naveen ChandraVega, Milena A.Jović-Jovičić, NatašaVelezmoro Sanchez, Carmen EloisaJu, JianzhuVenkatachalam, KarthikeyanJuhász, TamásVenkateswarlu, KattaJunaedi, SubaerVenkatraman, GopinathJúnior, JoãoVicaș, Laura GrațielaKacaniova, MiroslavaVindigni, VincenzoKadri, Nahrizul AdibVioleta, PurcarKakati, AbhijitVisa, AureliaKalmár, JózsefVlad-Bubulac, TachitaKalska-Szostko, BeataVlase, GabrielaKamaci, MusaVocanson, FrancisKamal, Muhammad ShahzadVozzi, GiovanniKamal, TahseenWadood, AbdulKamali, Ali RezaWakili, Karim GhaziKameneva, SvetlanaWalayat, NomanKamilah, HanisahWan, ZhiliKamińska-Dwórznicka, AnnaWanasundara, JanithaKamolz, Lars-PeterWandzik, IlonaKamperidou, VasilikiWang, BaominKan, ChiwaiWang, Chi-YunKanchan, Dinesh KumarWang, DehuiKandula, ManasaWang, GuijunKane, ShashankWang, HuanKania, DinaWang, Jian-WenKantaros, AntreasWang, JinKao, Chai-LinWang, JinguiKao, Chen-YuWang, JingxiaKaplan, GökhanWang, JunKarakus, SelcanWang, LiKarasinski, PawelWang, LiqiangKarayiannis, NikosWang, Peng-YuanKarlović, ZoranWang, QianKarpushkin, EvgenyWang, ShixueKasparavičienė, GiedrėWang, ShuKaul, AnkurWang, TingKaur, Chanchal DeepWang, WanheKaya, MükerremWang, WeihongKazachenko, AlexandrWang, XiaodongKaze, Cyriaque RodrigueWang, XingKean, ThomasWang, YaosongKeçili, RüstemWang, YingKehr, Nermin SedaWardhono, ArieKeskin, İlker ÇetinWardhono, Endarto YudoKhaled, MazenWaseem, AmirKhalid, Syed HaroonWei, GangKhalil, Islam A.Wei, JingKhan, AdnanWei, QinghuaKhan, Ikram UllahWekwejt, MarcinKhan, IrfanWia̧cek, Agnieszka EwaKhan, Nauman RahimWiench, RafałKhan, RajwaliWillenbacher, NorbertKhan, Shahid AliWimmer, MarkusKhan, Sher BahadarWinkler, Christina MariaKhan, SuhailWiśniewska, MałgorzataKhan, UmairWojciechowski, Jonathan P.Khan, ZiauddingWołowiec, StanisławKhandelwal, Nitesh KumarWong, BryanKhattab, TawfikWong, Leong SingKhayrutdinov, MaratWong, Siu Hong DexterKhoon, Lee TianWoźniak, MartaKhormali, AzizollahWu, HaitaoKhoshnevisan, KamyarWu, KekeKiiamov, AiratWu, ShijianKim, Gue-HyunWu, XinKim, Han SuWu, YahongKim, Ik-HwanWu, Yu-TseKim, KibeomXiao, YuKim, Sang BokXie, GangKim, Su-HwanXie, GuoxinKimura, TsuyoshiXie, LingxiaoKimura, YoshiharuXu, DekangKirilov, PlamenXu, HengyiKirilova, ElenaXu, RuijieKirubaharan, A. M. KamalanXu, YongKlabukov, IlyaXuan, TongtongKlimov, Viktor V.Xue, JiajiaKlimowicz, AdamXue, KunKljajević, LjiljanaYahya, EsamKlym, HalynaYajid, Muhamad Azizi MatKmita, AngelikaYan, Yong-BinKocak, FatmaYang, LetaoKocira, AnnaYang, YanyuKohli, KanchanYanina, Irina Yu.Kokkola, TarjaYazdian, FatemehKolesnikov, GennadiyYeh, Jui-MingKolhatkar, GitanjaliYildiz, GulcinKolotova, Daria S.Yilmaz, Catalina Natalia CheaburuKommineni, NagavendraYılmaz, EminKoncsag, Claudia IrinaYilmaz, OnurKondo, MizuhoYin, BohanKordestani, Soheila S.Yodyingyong, SupanKorolkov, Ilya V.Yoshizaki, YutaKörstgens, VolkerYosofvand, MohammadKorzhikova-Vlakh, EvgeniaYou, JiahuiKoseva, NeliYounes, HusamKótai, LászlóYounis, KaiserKotta, SabnaYoussef, Ahmed M.Kowalska, JolantaYu, FeiKozlova, EkaterinaYu, KunhaoKrajišnik, DaninaYu, Ollie YiruKrishna, ShankerYu, WeiKrishnakumar, Gopal ShankarYuan, WeihaoKruchinina, Natalia EvgenievnaYudin, VladimirKrystyjan, MagdalenaYusof, Nor AzahKrzan, MarcelZafar, AmeeduzzafarKuchler-Bopp, SabineZafar, Muhammad SohailKuckling, DirkZaharia, CatalinKudryashova, Elena V.Zaib, AurangKulbacka, JuulitaZaky, Zaky A.Kulkarni, DeepakZambrano, Maria De La LuzKulshrestha, SaurabhZang, ZhigangKumar, AnujZareNezhad, BahmanKumar, AvneeshZarepour, AtefehKumar, Govindan SureshZarrabi, AliKumar, PradeepZarrintaj, PayamKumar, RajeshZarzuela, RafaelKundu, DebashisZarzyka, IwonaKuo, Shu-JuiZavahir, SifaniKuo, Tsung-RongŻbikowska, AnnaKurkuri, MahaveerZdanowicz, MagdalenaKurlyandskaya, Galina V.Zeimaran, EhsanKusuma, HeriZelkó, RománaKusumastuti, YuniZeng, RuiKwon, Tae-YubZeng, XiaoweiLabus, KarolinaZha, ZhengbaoLai, Chin WeiZhang, AfangLaity, Peter R.Zhang, DaihuiLam, Kim-HungZhang, ErshuaiLange, JurgenZhang, HoujinLankalapallia, Ravi S.Zhang, HuLaokuldilok, ThunnopZhang, JianyongLapitsky, YakovZhang, JinchuangLarosa, ClaudioZhang, KaiLarrea-Wachtendorff, DominiqueZhang, KunxiLaurano, RossellaZhang, ShuyeLavaee, FatemehZhang, YanLe, PhungZhang, YifanLe, XiaoxiaZhang, YuLeal, João PauloZhang, ZhihongLee, Alvin Kai-XingZhao, ChangliLee, ChupingZhao, ChunLee, HyungseokZhao, DaweiLee, Mei-HwaZhao, JianzhangLee, Rong-HoZhao, KaiLee, See MunZhao, LeiLei, LanjieZhao, XinLeontopoulos, StefanosZheng, BingdeLeusheva, EkaterinaZheng, YafengLewandowicz, JacekZhengguo, WuLewandowska, KatarzynaZhong, TingLeyva-Porras, César C.Zhou, FeifeiLi, BangjingZhou, JinLi, BozhaoZhou, XuebingLi, ChenZhu, Qiu-JinLi, ChenyuZinina, OksanaLi, HuilingZou, HuibinLi, JinganZoughaib, MohamedLi, LeiZubavichus, YanLi, WeiZuccari, GuendalinaLi, XindaZuev, Yuriy FedorovichLi, Xueyu


